# Psychological Well-Being Among Older Chinese Migrants in Chiang Mai, Thailand: A Cross-Sectional Study on Structural and Psychosocial Resources

**DOI:** 10.3390/ejihpe15080154

**Published:** 2025-08-10

**Authors:** Xinyao Huang, Chawisa Suradom, Kelvin C. Y. Leung, Tinakon Wongpakaran, Rewadee Jenraumjit

**Affiliations:** 1 Master of Science Program (Mental Health), Multidisciplinary Interdisciplinary School, Chiang Mai University, Chiang Mai 50200, Thailand; xinyao_huang@cmu.ac.th (X.H.); chawisa.s@cmu.ac.th (C.S.); tinakon.w@cmu.ac.th (T.W.); 2 Department of Psychiatry, Faculty of Medicine, Chiang Mai University, Chiang Mai 50200, Thailand; 3 Specialty of Psychiatry, Faculty of Medicine and Health, University of Sydney, Sydney, NSW 2006, Australia; kelvin.leung@sydney.edu.au; 4 Department of Pharmaceutical Care, Faculty of Pharmacy, Chiang Mai University, Chiang Mai 50200, Thailand

**Keywords:** well-being, older migrants, active aging, sense of mastery, loneliness, social support, cross-cultural adaptation, social determinants of health

## Abstract

Despite the growing number of older adults engaging in voluntary migration, there is a lack of knowledge about their psychological well-being in cross-cultural contexts. This cross-sectional study investigated factors associated with psychological well-being among older Chinese migrants residing in Chiang Mai, Thailand. Between December 2024 and February 2025, 204 Chinese migrants aged 60 and above who had resided in Chiang Mai for at least six months participated in a survey in Chinese. The survey measured sociodemographic and psychosocial factors including perceived health, income, marital status, number of co-residing family members, social support, acculturative stress, sense of mastery, and loneliness. Multiple regression analysis showed that gender (female) (*p* = 0.006), better perceived health status (*p* = 0.021), higher income (*p* = 0.007), more co-residing family members (*p* = 0.037), a greater sense of mastery (*p* = 0.009), and lower levels of loneliness (*p* < 0.001) were each independently associated with better psychological well-being. In contrast, neither general family support nor acculturative stress was a statistically significant predictor. These findings highlight the significant roles of financial security, family co-residence, personal empowerment, and social connectedness in shaping overall well-being. Strategies to improve psychological well-being in this population should focus on strengthening emotional connectedness, supporting the development of meaningful family and social relationships, and supporting economic stability.

## 1. Introduction

As life expectancy increases and birth rates decline worldwide (World Health Organization, n.d.), a growing number of older adults remain active well into later life, and some opt to relocate internationally (He & Sasiwongsaroj, 2024; Sasiwongsaroj & Husa, 2022). Unlike labor migrants or refugees, older adults who migrate after retirement typically make this transition voluntarily, value their independence, and are primarily motivated by family ties, lifestyle preferences, or the pursuit of comfort (He & Sasiwongsaroj, 2024; Sasiwongsaroj & Husa, 2022). Recent migration reports indicate that the number of foreigners residing in Thailand on a retirement visa has grown significantly—from 72,969 in 2018 to 126,654 in 2023, representing an increase of nearly 74% (United Nations Network on Migration in Thailand, 2024). While national data do not specify the proportion of Chinese nationals, estimates suggest that approximately 110,000 to 130,000 Chinese migrants currently reside in Thailand, including retirees (Lertpusit, 2023). Immigration records indicate that over 4000 Chinese nationals annually extended their retirement visas between 2020 and 2022 (Lertpusit, 2023), reflecting a steady pattern of long-term settlement. These migrants have contributed not only to the local economy—particularly in the real estate, hospitality, and service sectors—but also to the cultural vibrancy of areas with large Chinese-speaking communities (OECD & International Labour Organization, 2017). Given this trend, Thailand—particularly Chiang Mai—has become an attractive destination for many older Chinese migrants due to its geographical proximity, cultural familiarity, and affordable healthcare (Sirivadhanawaravachara, 2024). Many of these migrants live independently and maintain strong social ties within Chinese-speaking communities, emphasizing both a desire for dignity and a focus on psychological well-being in later life (Jiaqi, 2022).

Despite these apparent advantages, little is known about how older Chinese migrants emotionally adapt to their new cultural environment. Much of the existing research has emphasized either physical health or available services for migrants, while the psychological aspects of adjusting to a new culture remain understudied (Hawkins et al., 2022). This gap is particularly notable for older adults who migrate voluntarily after retirement—a group often overlooked in both international migration and aging research (Savaş et al., 2023). The experience of growing older abroad presents unique challenges and opportunities (Tang & Zolnikov, 2021). Older migrants must navigate new environments often with limited institutional support, making the preservation of psychological well-being both complex and critical (Anantapong et al., 2024). Subjective well-being, which encompasses overall emotional state and life satisfaction, offers a useful lens through which to understand this adjustment process (Hendriks & Bartram, 2019). Recent studies have highlighted how emotional strain can emerge even among seemingly well-integrated older migrants, particularly when facing intergenerational tensions, limited language proficiency, or a perceived loss of family roles (Dong et al., 2014; Fang et al., 2014; Lai et al., 2020). For instance, studies among older migrants in Canada and the Netherlands have emphasized the role of neighborhood belonging and access to culturally appropriate services in shaping psychological well-being (Conkova et al., 2024; Lai et al., 2020). Similarly, research in South Korea and Australia has found that acculturative stress, intergenerational conflict, and perceived discrimination are major predictors of emotional distress in aging migrant populations (Joshi et al., 2025; Tran et al., 2022).Yet, research from Southeast Asia remains scarce, underscoring the need for more geographically diverse evidence (Bhatia et al., 2024).

Multiple factors influence well-being in later life. Psychosocial and structural resources such as good physical health, financial stability, and being married or partnered are consistently linked to better emotional outcomes (Lu et al., 2023). Other environmental conditions—such as language proficiency, living arrangements, and length of residence—may also relate to adjustment. For example, a limited language ability may restrict access to services and emotional expression, especially for those with low education levels or strong dialect backgrounds (Montemitro et al., 2021). Longer stays in the host country may allow for greater cultural familiarity and social networks, while living with family members may offer emotional support or, at times, create stress due to intergenerational tensions (Q. Ren & Treiman, 2015; Song & Chen, 2020).

In addition to structural conditions, personal psychological resources also play a role. Social support and a sense of mastery are often cited as important buffers against stress (Guo et al., 2018a; Lai et al., 2020). Cross-cultural studies in Sweden, Japan, and the United States have shown that such internal resources play a compensatory role when external support is limited, especially in later-life migration contexts (Anantapong et al., 2024; Magnusson, 2024). A strong sense of mastery, which is defined as the belief in one’s ability to manage life events, may help individuals to maintain emotional stability when external conditions fluctuate (Bian et al., 2024). However, such internal resources may function differently depending on the individual’s environment and the intensity of external demands. Conversely, factors such as acculturative stress and loneliness frequently diminish well-being. Older migrants may experience psychological strain when adjusting to unfamiliar social norms and systems (Choi & Kang, 2025). Even for those who appear autonomous and socially active, the interplay of these factors may influence how individuals adjust psychologically when adapting to a new country (Hendriks & Bartram, 2019).

Understanding how older Chinese migrants maintain well-being during cultural adaptation provides valuable insights into the psychological dynamics of aging abroad. This study draws on the World Health Organization’s Active Ageing Framework (World Health Organization, 2002), which highlights the three pillars essential for aging well: security, participation, and health. The framework recognizes older adults as active contributors to society who could retain their engaged, independent, and mobile qualities reflected in our study participants (Sasiwongsaroj & Husa, 2022). The WHO-5 Well-Being Index captures these constructs, focusing on positive emotional functioning, unlike symptom-based scales for depression or anxiety (World Health Organization Regional Office for Europe, 1998). Despite residing in a foreign context, many of our subjects maintained autonomy, physical activity, and social connectedness, consistent with active aging ideals.

To more clearly reflect the Active Ageing Framework, we categorized the study variables under its three core pillars: health, security, and participation (World Health Organization, 2002). The “health” dimension includes perceived health status and language proficiency, as these affect both physical capability and access to services. The “security” domain covers monthly income, length of residence, visa status, and health insurance, reflecting migrants’ economic and legal stability (Hawkins et al., 2022; Jacobsen et al., 2023). The “participation” pillar encompasses social support, sense of mastery, co-residing family members, and loneliness, all of which relate to autonomy, relational engagement, and perceived connection. Acculturative stress and age, while not directly aligned with the original WHO pillars, serve as contextual factors influencing how older migrants experience aging abroad (Flynn et al., 2023; Johansson et al., 2022; Narushima et al., 2018).

Guided by this framework, the present study aims to explore the psychosocial factors associated with psychological well-being among older Chinese migrants living in Chiang Mai, Thailand. Specifically, we address the following research questions: (1) What is the overall level of psychological well-being among this population? (2) How are socio-economic and psychosocial resources associated with well-being? We hypothesize that greater income, better self-rated health, being married or partnered, higher levels of social support and mastery, and lower levels of loneliness and acculturative stress will be significantly associated with higher psychological well-being. These hypotheses are informed by the existing literature on aging, migration, and mental health in later life, as discussed in the preceding sections.

## 2. Materials and Methods

### 2.1. Study Design and Participants

This cross-sectional study was conducted in Chiang Mai Province, Thailand from December 2024 to February 2025. Participants were older Chinese migrants living in Thailand. The inclusion criteria were as follows: (1) older people who migrated to Thailand for a long stay and have been in Thailand for more than 6 months, (2) migrated to Thailand after 60 or older, and (3) ability to hear, read, write, and communicate sufficiently in Chinese. The exclusion criteria included incomplete responses or a history of diagnosed mental disorders, cerebrovascular disease, Parkinson’s disease, dementia, disability, bedridden, or brain injury.

### 2.2. Sample Size and Sampling Technique

The sample size was calculated using the formula for estimating a population proportion with specified precision (Lwanga et al., 1991). The calculation assumed a reasonable estimated prevalence of mental health symptoms based on prior studies in similar populations, with a 95% confidence level and a 5% margin of error. The minimum required sample was 246. To allow for non-response, the target sample was increased to 270. A total of 204 complete responses were included in the final analysis. Given the dispersed nature of the target population and the absence of formal registries, a convenience sampling technique was employed. Although this approach limits the generalizability of the findings, it was a culturally appropriate and practical method for engaging a group that is both underrepresented and difficult to reach.

**Figure 1:**
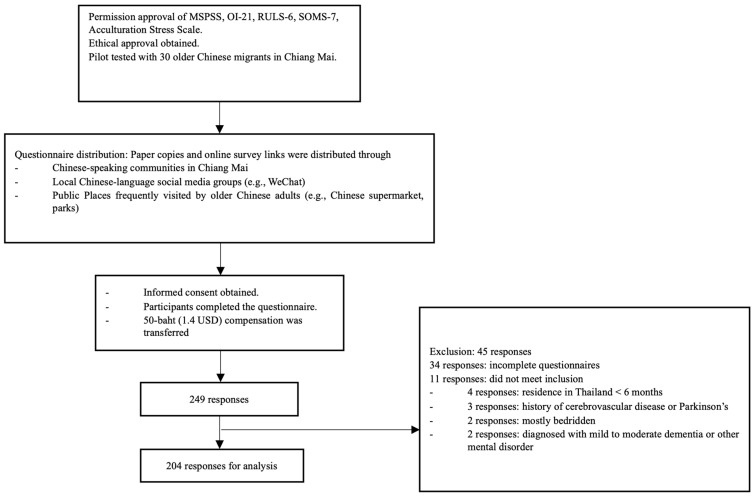
Flow chart of the study.

As shown in Figure 1, the flowchart begins with the ethical and procedural approvals. A pilot test was conducted with 30 older Chinese migrants in Chiang Mai. Following this, recruitment and questionnaire distribution were carried out using convenience sampling; paper and online survey forms were distributed throughout Chinese-speaking community organizations in Chiang Mai, local Chinese-language social media groups such as WeChat, and community venues frequented by the target population, including Chinese supermarkets and parks. Informed consent was obtained from all participants before they completed the questionnaire. The study received a total of 249 responses. Of these, 34 responses were excluded due to incomplete questionnaires, and an additional 11 were excluded for not meeting the eligibility criteria. This process resulted in a final analytic sample of 204 complete and eligible responses included for analysis.

### 2.3. Questionnaires and Measurements

#### 2.3.1. Sociodemographic Information

The questionnaire included age, gender, marital status, income, education, co-residence with children, number of household members, perceived health status (measured using a single self-rated item: ‘How would you describe your physical health?’, with five response options ranging from ‘very poor’ to ‘very good’), length of stay in Thailand, language proficiency (Chinese, Thai, and English), and migration motivations.

Prior to formal data collection, a pilot test was conducted among older Chinese migrants in Chiang Mai to assess the applicability of the combined questionnaire. The pilot study demonstrated acceptable internal consistency across all scales, with Cronbach’s alpha coefficients ranging from 0.76 to 0.89: MSPSS (α = 0.89), Acculturative Stress Scale (α = 0.80), SOMS-7 (α = 0.78), RULS-6 (α = 0.76), and WHO-5 (α = 0.82). These results yielded an overall Cronbach’s alpha of 0.85 for the full questionnaire, indicating good internal consistency. These findings suggest preliminary support for the construct validity of the scales in this population.

#### 2.3.2. Perceived Social Support

The Multidimensional Scale of Perceived Social Support (MSPSS) comprises 12 items that measure support from family, friends, and significant others. Items are rated on a 7-point scale (1 = very strongly disagree, 7 = very strongly agree). In the Chinese version, the Cronbach’s alpha was 0.89 (Ng et al., 2022; Zimet et al., 1988). In this study, the Cronbach’s alpha was 0.944.

#### 2.3.3. Loneliness

The 6-item Revised UCLA Loneliness Scale (RULS-6) assesses subjective feelings of loneliness. Responses range from 1 (never) to 4 (always), with higher scores indicating greater loneliness. The Cronbach’s alpha was reported as 0.83, indicating good internal consistency (Wongpakaran et al., 2020). In this study, the Cronbach’s alpha was 0.878.

#### 2.3.4. Sense of Mastery

The 7-item Sense of Mastery Scale (SOMS-7) measures an individual’s perceived control over their life. Responses are measured on a 4-point scale (1 = strongly disagree, 4 = strongly agree). Higher scores indicate stronger mastery. In the Chinese version, the Cronbach’s alpha was 0.84 (Yu & Zou, 2008). In this study, the Cronbach’s alpha was 0.663.

#### 2.3.5. Acculturative Stress

Acculturative stress was assessed using the short version of the scale developed for the Chinese community of Kolkata, which consists of 10 items rated from 1 (strongly disagree) to 5 (strongly agree). Higher scores reflect more stress. The Cronbach’s alpha was 0.87 (Biswas, 2022). In this study, the Cronbach’s alpha was 0.903.

#### 2.3.6. Psychological Well-Being

The WHO-5 Well-Being Index comprises five items that measure emotional well-being over the previous two weeks. Each item is scored from 0 (never) to 5 (always), with total scores ranging from 0 to 25. Higher scores indicate better well-being. A raw score below 13 (or a percentage score below 50%) has been suggested as a cut-off for poor mental well-being and as an indication for further assessment of possible mental health conditions (World Health Organization Regional Office for Europe, 1998). The Cronbach’s alpha was 0.85 (Fung et al., 2022). In this study, the Cronbach’s alpha was 0.870.

### 2.4. Data Analysis

All data were analyzed using IBM SPSS software (Version 26.0, IBM Corp, Armonk, NY, USA). Descriptive statistics (means, standard deviations, and frequencies) were used to summarize participants’ sociodemographic characteristics and psychological variables. Cronbach’s alpha coefficients were calculated to assess the internal consistency of each scale, with reverse scoring applied where necessary. To examine differences in well-being, chi-square tests were used for categorical variables, and independent-sample t-tests were applied to compare mean well-being scores across binary groups. All hypothesized variables were entered into a multiple linear regression model to identify predictors of well-being. The regression results included several key indicators: regression coefficients (β), which reflect the direction and magnitude of predictor effects; standard errors; R-squared and adjusted R-squared values, which assess model fit, and the proportion of variance explained. Diagnostic analyses were conducted to ensure the appropriateness and robustness of the regression model. Outliers were addressed by excluding cases with standardized residuals exceeding ±3. The assumptions of normality, linearity, and homoscedasticity were assessed visually using Q–Q plots, histograms of residuals, and residual scatterplots. Multicollinearity was evaluated using the Variance Inflation Factor (VIF), with all values below 3 indicating no substantial multicollinearity among predictors. Statistical significance was set at a two-tailed *p*-value of <0.05.

## 3. Results

### 3.1. Sociodemographic Variables

Most participants were in the 60–69 age group (76.5%), with fewer aged 70 or above (23.5%). Slightly more than half were female (52.9%), while males comprised 47.1% of the sample. The majority of respondents reported a high level of education (73.0%). Three-quarters (75.5%) were married or in a partnered relationship. In terms of health, 58.8% perceived their health as good or very good, while 41.2% rated it as average or poor. Slightly more than half of participants (54.9%) reported a low monthly income, with the remaining 45.1% in the moderate-to-high income category (Table 1).

### 3.2. Psychological Measures

Table 2 describes the participants’ psychological measures. The average perceived social support score was relatively high (M = 60.72, SD = 14.50). The mean acculturation stress score was moderate (M = 43.61, SD = 12.00), as were the mean scores for sense of mastery (M = 22.31, SD = 4.38) and loneliness (M = 13.02, SD = 4.30). The average well-being score, measured using the WHO-5, indicated a moderate level of positive mental health (M = 15.98, SD = 4.43).

### 3.3. Participants’ Well-Being Profiles

Table 3 presents the primary outcome of the study, the WHO-5 Well-Being Index. Participants reported the highest average score for feeling calm and relaxed, followed by feeling cheerful and in good spirits. The lowest average score was for daily life being filled with things that interest them. The total mean well-being score was 15.98 (SD = 4.43), indicating a moderate level of positive mental health among participants.

### 3.4. Pearson Correlation Analysis of Key Variables

Table 4 presents the intercorrelations among key sociodemographic, language, psychosocial, and outcome variables. The main outcome, well-being, was positively correlated with monthly income, Thai proficiency, length of residence, social support, and sense of mastery, and negatively correlated with acculturation stress and loneliness. Notably, loneliness and acculturation stress showed the strongest negative correlations with well-being, while sense of mastery and social support were among the strongest positive correlates. Several key variables, such as monthly income and sense of mastery, were also related to other psychosocial factors, highlighting the interconnected nature of these predictors in relation to mental health.

### 3.5. Predictors of Well-Being

Table 5 summarizes the multiple regression analysis of predictors of psychological well-being, accounting for the interrelations among variables that were previously identified in bivariate correlation analysis. Consistent with the earlier correlations, loneliness was the strongest negative predictor of well-being (β = −0.582, *p* < 0.001), and sense of mastery was a significant positive predictor (β = 0.160, *p* = 0.009). Female gender, greater number of co-residing family members, better perceived health, and higher monthly income also significantly predicted higher well-being. Notably, while variables such as social support, acculturative stress, and Thai proficiency showed significant correlations with well-being, they did not independently predict well-being after controlling for other variables in the regression model. These results highlight the central role of loneliness, sense of mastery, and several sociodemographic factors in shaping psychological well-being among the participants.

To evaluate model reliability, diagnostic checks revealed that standardized residuals were predominantly within ±2, indicating no extreme outliers. The Q–Q plot demonstrated that residuals closely aligned with the normality line, with only minor deviations in the upper tail, while the histogram suggested an approximately normal distribution. The residuals-versus-fitted plot showed no discernible pattern, supporting the assumption of homoscedasticity. Variance Inflation Factor (VIF) values ranged from 1.07 to 2.90, indicating no serious concerns regarding multicollinearity. Overall, the model accounted for a substantial proportion of variance in positive mental health, with no notable violations of regression assumptions.

## 4. Discussion

This study examined factors associated with psychological well-being among older Chinese migrants in Chiang Mai, Thailand. The results demonstrated that, among females, the number of co-residing family members, perceived health status, monthly income, sense of mastery, and loneliness were significantly related to the outcome.

Loneliness was the most powerful negative predictor; previous studies have found that loneliness significantly undermines both emotional and physical health among older migrants (Lai et al., 2020). Loneliness is increasingly recognized not only as an emotional state, but as a core social determinant of health, particularly in aging populations living in unfamiliar environments (Kearns et al., 2015; VanderWeele et al., 2012). Even in close-knit ethnic communities, emotional loneliness can persist, since being physically close does not guarantee emotional closeness or supportive relationships (Bilecen & Vacca, 2021). This is a consistent global finding: social isolation, even within a linguistically similar community, is detrimental to both emotional and physical health. For older migrants, disrupted friendships and networks, loss of ancestral community, or emotional gaps within the family may heighten feelings of loneliness (Pan et al., 2023). Despite living in Chinese-speaking communities, many participants still reported feeling emotionally disconnected. Indeed, recent research showed that the key predictors of loneliness are not cultural origin, but individuals’ coping capacity, relationship opportunities, and the perceived quality of the social environment. Higher social capital, active coping strategies, and relationship mobility serve as protective factors, while discrimination and ageism contribute to loneliness regardless of migration status (Pan et al., 2023).Therefore, efforts to enhance psychological well-being should focus on trusting relationships, rather than relying solely on proximity or cultural familiarity (Umberson & Montez, 2010).

As expected, a higher sense of mastery—feeling capable and in control of life circumstances—was a significant positive predictor of well-being. Research shows that mastery is closely linked to both mental and physical health. Migrants who believe that they can influence what happens in their lives are more likely to handle challenges, manage stress, and avoid problems that harm health. Feeling a sense of control helps them to stay active, seek help when needed, and remain engaged in social activities, all of which support emotional stability. A strong sense of mastery may also encourage older migrants to form new social networks, learn the local language, and proactively access resources that ease their adjustment, further sustaining well-being. By contrast, when older migrants feel helpless or think they have little control, they may experience more stress and isolation, which may be associated with lower well-being (Chaze & Robson, 2014).

For socio-demographic and related factors, gender was found to be a significant predictor. In some older migrant communities, women may engage more actively in social networks, mutual aid groups, or family care, which could boost emotional support and well-being (Shin & Park, 2023). Alternatively, women may be more likely to seek help or participate in group activities, which can contribute to greater subjective well-being (Nayak et al., 2025). These patterns may reflect broader gender role expectations in later life and during migration. According to gendered migration frameworks, women’s social positioning often allows for greater flexibility in rebuilding interpersonal ties and accessing care-based roles that reinforce identity and belonging (Cabieses et al., 2023; Fathi & Ní Laoire, 2024). Conversely, in some contexts, men’s traditional roles may not translate as well in the host environment. This, combined with strong adherence to conventional masculine norms that discourage emotional expression and help-seeking, can increase psychological distress and contribute to lower well-being among men (Mokhwelepa & Sumbane, 2025). Additionally, men may face role loss or diminished status in unfamiliar settings, especially if their prior occupations or identities are less relevant after migration (Zhu & Chan, 2025).

Co-residence may buffer emotional stress, offer daily companionship, and provide practical support for older adults, particularly migrants who might otherwise experience isolation. The extended family model may facilitate the continuity of cultural practices and shared understanding. In cultures valuing filial piety, living with adult children can help to reduce loneliness, even during stressful situations (Jiao et al., 2024). Moreover, co-residence may provide older adults with a greater sense of security and belonging, allowing them to maintain confidence in daily life and adapt more effectively to cultural differences. This shared living arrangement can create conditions that allow for intergenerational exchanges that support emotional resilience and help sustain overall well-being (Carson, 2024). Regular emotional interactions and financial support from adult children can further enhance positive emotions and promote social participation (K. Ren et al., 2025).

Beyond these instrumental and emotional functions, co-residence in collectivistic or familistic cultures may also serve as a symbolic marker of relational harmony and intergenerational unity. The cultural expectation of staying close to family, both physically and emotionally, may help to explain how older adults evaluate their well-being. As shown in recent cross-cultural studies, the meaning and psychological impact of living arrangements may differ substantially depending on cultural norms, especially during later-life transitions such as the empty-nest period. (Hartanto et al., 2024)

Financial stability was positively associated with well-being, in line with the Social Determinants of Health model (Bhatia et al., 2024). Participants with higher income reported better outcomes, likely because financial resources reduce daily stress and support a sense of independence and control. For older migrants without formal entitlements, income may also buffer uncertainties in a new environment (Gildner et al., 2019).

Perceived health status was also a significant factor in well-being. This aligns with a wealth of research showing that both objective and perceived health strongly influence well-being in older adults, supporting autonomy, social participation, and optimism. Participants reporting good or excellent health tended to have higher levels of well-being. Similar patterns have been observed in older adult populations in different contexts (Zhang et al., 2022). For migrants, good health may reduce the need for unfamiliar healthcare services and support greater self-sufficiency (Jang et al., 2020).

Although several variables—including marital status, language proficiency, and perceived social support—are often cited as key determinants of well-being in the literature, they did not reach statistical significance in this sample. Several possible explanations may account for this. First, their effects may be indirect, operating through more proximal factors such as loneliness, mastery, or financial stability. For instance, being married or having stronger language skills might enhance well-being primarily by reducing loneliness or promoting a sense of control, rather than exerting a direct influence (Guruge & Sidani, 2023; Pan et al., 2023). In our sample, most participants lived in Chinese-speaking environments and maintained their primary contact within ethnic communities. This may have reduced the functional relevance of Thai or English proficiency in daily interactions, thereby weakening its direct association with well-being. Similarly, while many respondents reported being married or partnered, the emotional quality of those relationships was not assessed, suggesting that marital status alone may not fully capture its psychological benefits. Second, many psychosocial variables in our model are conceptually and empirically interconnected. A supportive social environment may enhance perceived control (sense of mastery), while mastery may, in turn, facilitate relationship-building and help-seeking. Likewise, language proficiency may influence well-being by increasing participation or decreasing social isolation. These dynamics suggest that some variables may act as mediators, moderators, or suppressors of others, potentially obscuring their individual effects (Guo et al., 2018b; Lee et al., 2022). Third, some effects may have been context dependent. For example, the social buffering hypothesis posits that the protective role of social support is most evident under conditions of elevated stress or vulnerability, which may not have been uniformly present in our sample (Kim et al., 2022; Vila, 2021). Finally, the lack of significant associations may also reflect sample-specific characteristics, such as a relatively homogeneous group in terms of retirement status, education, or income. This could reduce variability and mask effects that are detectable in more heterogeneous populations. To better understand these non-significant results, future research should move beyond examining each variable in isolation. Methods such as structural equation modeling could help to identify indirect effects, mediating pathways, and suppression effects among psychosocial predictors. This would provide a more nuanced understanding of how factors such as social support, mastery, and language proficiency interact to shape well-being in later life, particularly within the unique cultural and migration contexts of older adults. 

### 4.1. Practical Implications

These findings highlight the importance of both structural resources (e.g., income stability or access to care) and psychological strengths (e.g., a strong sense of mastery) in sustaining well-being among older migrants in Thailand. In particular, loneliness emerged as a key concern and should be prioritized in future interventions. Accessible healthcare is an essential foundation in this context, but its benefits are most effective when paired with emotionally supportive environments. Co-residence or family contact may help, but must involve genuinely caring relationships. Evidence from recent studies suggests that culturally sensitive programs (Pocock et al., 2020), including peer-based support (Lai et al., 2020), community engagement initiatives (Alfieri et al., 2019), and intergenerational mentoring (Juris et al., 2022; Sneed & Chan, 2023), may help to reduce social isolation while enhancing autonomy, self-efficacy, and emotional resilience among aging migrant populations. Services should also improve health access through native-language check-ups, trained mediators, and simplified systems (Kosiyaporn et al., 2020).

These findings may contribute to ongoing discussions around the WHO’s Active Ageing Framework by highlighting the need to address emotional and cultural vulnerabilities among older migrants (World Health Organization, 2021). In countries that increasingly receive retirees from abroad, such as Thailand and other aging destinations in Asia, the current reception models often lack formal structures for psychological support or social integration (United Nations Network on Migration in Thailand, 2024). This study underscores the importance of embedding emotional well-being and culturally responsive care into aging and migration policies. 

### 4.2. Strengths and Limitations

This study is the first to specifically investigate psychological well-being and its associated factors among older Chinese migrants in Thailand. This group has been rarely addressed in the existing research. The findings offer valuable insights into this issue. Nonetheless, several limitations must be acknowledged. The cross-sectional design does not allow for causal inferences. The sample, drawn only from Chiang Mai, limits the generalizability of the results to older migrants in other areas. All data were self-reported, which may introduce recall or social desirability bias. Recruitment proved to be challenging, resulting in a smaller-than-anticipated sample size and potentially excluding individuals who are more isolated or less willing to participate. There may also be self-selection bias, as individuals with higher well-being or stronger social ties might have been more willing to participate. Additionally, some culturally specific factors, such as filial piety, which may influence co-residence and intergenerational relationships, were not included in this study. Future studies would benefit from broader geographic coverage, larger and more varied samples, comparative groups, and longitudinal approaches to better capture the dynamic and evolving nature of psychological well-being among older migrant populations. It is also important to recognize the potential conceptual overlap between some predictors and the well-being outcome. For example, measures of loneliness and general well-being both contain substantial affective content, and their strong intercorrelation may reflect shared variance. This overlap suggests that variables such as loneliness may function not merely as unidirectional predictors, but also as mediators, moderators, or outcome-related constructs within a complex, multidimensional framework. Future studies should consider these analytic and theoretical complexities, applying advanced modeling techniques and collinearity diagnostics to disentangle these relationships and better clarify the unique contributions of proximal and distal determinants of well-being.

## 5. Conclusions

This study provides new evidence that economic stability, co-residence with family members, sense of mastery, and especially reduced loneliness are key contributors to psychological well-being among older Chinese migrants in Chiang Mai, Thailand. These findings reinforce the importance of designing culturally sensitive interventions that not only address material needs, but also support emotional connection, autonomy, and meaningful engagement in later life. In practical terms, this is tailored to older migrant populations. The study also highlights the need for receiving countries—particularly those attracting large numbers of retirement migrants—to recognize older migrants as a distinct policy group requiring targeted inclusion strategies. Future studies should adopt longitudinal or mixed-method designs to explore causal pathways and dynamic changes over time. Comparative research with other migrant groups in Thailand (e.g., Japanese retirees) or across regions would also enrich our understanding of how cultural background, policy context, and migration experience inform our understanding of later-life well-being. 

## Figures and Tables

**Table 1:** Sociodemographic characteristics of participants (*n* = 204).

Variable	n (%)
Age group	
60–69	156 (76.5)
≥70	48 (23.5)
Gender	
Female	108 (52.9)
Male	96 (47.1)
Education	
High	149 (73.0)
Low	55 (27.0)
Marital status	
Married/Partnered	154 (75.5)
Not married	50 (24.5)
Perceived health status	
Good/Very good	120 (58.8)
Average/Poor	84 (41.2)
Monthly income	
Moderate–High income	92 (45.1)
Low income	112 (54.9)

Note. High education = high school, vocational school, bachelor’s degree, or above; Low = middle school or below; Moderate–High income = monthly income ≥ 20,000 THB; Low income = < 20,000 THB; and “Married/Partnered” includes those currently married or cohabiting.

**Table 2:** Descriptive statistics of key psychological variables (*n* = 204).

Variables	
Scores of Psychological Measures	Mean (SD)
Perceived social support (range 12–84)	60.72 (14.50)
Acculturation stress (range 16–76)	43.61 (12.00)
Sense of mastery (range 10–32)	22.31 (4.38)
Loneliness (range 6–24)	13.02 (4.30)
Well-being (range 3–25)	15.98 (4.43)

Note: Values represent means and standard deviations (SDs). Higher scores indicate higher levels of the respective construct.

**Table 3:** Item-level scores for the WHO-5 Well-Being Index (*n* = 204).

WHO-5 Item	Mean	Standard Deviation
1. I have felt cheerful and in good spirits	3.24	1.10
2. I have felt calm and relaxed	3.39	0.97
3. I have felt active and vigorous	3.10	1.14
4. I woke up feeling fresh and rested	3.17	1.09
5. My daily life has been filled with things that interest me	3.08	1.12
Total score (range 0–25)	15.98	4.42

**Table 4:** Pearson correlation matrix of key variables.

Variables	1	2	3	4	5	6	7	8	9	10	11	12
1.Age	1											
2. Co-residing family no.	−0.045	1										
3. Monthly income	0.055	0.032	1									
4. Chinese proficiency	−0.127	−0.136	−0.077	1								
5. Thai proficiency	0.028	0.292 **	0.119	−0.087	1							
6. English proficiency	0.080	0.009	0.160 *	−0.116	0.213 **	1						
7. Length of residence	0.080	0.275 **	0.129	0.023	0.473 **	0.166 *	1					
8. Social support	0.119	0.052	0.131	0.037	0.179 *	0.162 *	0.294 **	1				
9. Acculturation stress	−0.002	−0.072	−0.392 **	−0.026	−0.070	−0.112	−0.170 *	−0.325 **	1			
10. Sense of mastery	0.075	0.016	0.268 **	0.037	0.024	0.127	0.051	0.354 **	−0.689 **	1		
11. Loneliness	−0.073	−0.138 *	−0.273 *	−0.049	−0.179 *	−0.152 *	−0.170 *	−0.389 **	0.735 **	−0.690 **	1	
12. Well-being	−0.050	0.099	0.368 **	0.063	0.138 *	0.110	0.155 *	0.326 **	−0.601 **	0.561 **	−0.704 **	1

Note. Pearson correlation coefficients are reported. * *p* < 0.05, ** *p* < 0.01 (two-tailed).

**Table 5:** Multiple regression predicting well-being.

Predictor	B	SE	β	*p*-Value
(Constant)	24.436	2.552		<0.001
Gender (female)	0.956	0.344	0.116	0.006
Age	0.531	0.370	0.062	0.153
District of residence	−0.004	0.033	−0.005	0.914
Marital status	−0.423	0.462	−0.044	0.361
Education level	0.151	0.162	0.047	0.351
Living with children	−0.148	0.459	−0.018	0.747
Number of co-residing family members	0.597	0.283	0.113	0.037
Perceived health status	0.637	0.273	0.110	0.021
Chinese proficiency	−0.644	0.447	−0.062	0.151
Thai proficiency	−0.232	0.204	−0.069	0.255
English proficiency	0.369	0.199	0.099	0.066
Monthly income	0.642	0.234	0.132	0.007
Health insurance	−0.107	0.214	−0.024	0.616
Length of residence	−0.132	0.215	−0.031	0.540
Visa type	0.816	0.509	0.073	0.111
Perceived social support	−0.009	0.013	−0.034	0.481
Acculturative stress	−0.020	0.025	−0.056	0.414
Sense of mastery	0.156	0.060	0.160	0.009
Loneliness	−0.578	0.069	−0.582	<0.001
R^2^ = 0.722, Adjusted R^2^ = 0.691				

Note. B = unstandardized coefficient; SE = standard error; β = standardized coefficient; and *p*-value = significance level.

## Data Availability

The data presented in this study are available upon request from the corresponding author. Due to ethical restrictions, they are not publicly available.
